# Case Report: Eccentric purulent pericarditis treated by PTCA guidewire-based pericardiocentesis and intrapericardial alteplase

**DOI:** 10.3389/fcvm.2025.1660781

**Published:** 2025-08-05

**Authors:** Can Li, Chun-Chang Qin, Yue Jiang, Lin Hou

**Affiliations:** ^1^ Department of Cardiovascular Medicine, Cardiovascular Research Center, The First Affiliated Hospital of Chongqing Medical University, Chongqing, China; ^2^ Department of Cardiovascular Medicine, The Fifth People’s Hospital of Chongqing, Chongqing, China

**Keywords:** PTCA guidewire-based pericardiocentesis, eccentric purulent pericarditis, fibrinolysis, pericardial effusion, cross-disciplinary application of medical devices

## Abstract

**Background:**

Purulent pericarditis is an infectious condition characterized by purulent pericardial effusion.

**Case presentation:**

In this case report, we present a 60-year-old male admitted with intermittent chest pain and fever, ultimately diagnosed with primary purulent pericarditis. Imaging revealed an eccentric loculated pericardial effusion, posing significant challenges for conventional pericardiocentesis. In this context, we innovatively employed a percutaneous transluminal coronary angioplasty (PTCA) guidewire-based pericardiocentesis technique, achieving successful catheter placement followed by intrapericardial administration of 5 mg alteplase. During follow-up, the patient achieved complete clinical recovery.

**Conclusions:**

This report introduces a PTCA guidewire-based pericardiocentesis approach, which safely addresses complex eccentric loculated pericardial effusions. Combined with low-dose intrapericardial alteplase, drainage efficacy was enhanced, pericardial adhesions were reduced, and invasive pericardiectomy could be avoided. This minimally invasive technique provides a viable alternative to surgery for high-risk patients.

## 1 Introduction

Purulent pericarditis (PP) is defined as neutrophil-dominant pericardial effusion caused by bacterial, fungal, or parasitic infections. This condition is associated with high mortality, primarily due to cardiac tamponade and septic shock (1). Widespread antibiotic use has reduced its prevalence to 1% of pericardial diseases; however, the mortality rate remains significant, with only 85% of patients surviving despite comprehensive treatment (2). Patients with PP are at risk of developing constrictive pericarditis secondary to pericardial adhesions and fibrosis. Fibrinolytic agents may dissolve fibrin strands, reduce adhesions, enhance drainage, and potentially mitigate this complication (3). We present a case of PP in which echocardiography revealed a large loculated effusion in the right posterior pericardial cavity. Given the high risk of conventional pericardiocentesis, percutaneous transluminal coronary angioplasty (PTCA) guidewire-based pericardiocentesis was successfully performed, followed by intrapericardial administration of low-dose alteplase, ultimately achieving complete resolution.

## 2 Case description

A 60-year-old male with a history of hypertension and diabetes mellitus was admitted for intermittent chest pain and fever lasting two days. The patient experienced retrosternal pain that worsened with deep inspiration and supine positioning and radiated to the shoulder and back, along with a fever of 38.7 °C. Initial electrocardiography (ECG) demonstrated ST-segment elevation (0.1–0.3 mV) in the lateral, inferior, and extensive anterior walls (Supplementary Figure S1). Cardiac biomarkers remained within normal limits, and transthoracic echocardiography (TTE) was unremarkable. Laboratory tests revealed leukocytosis [white blood cell (WBC) count 10.99 × 10^9^/L, neutrophils 83.8%]. Coronary computed tomography angiography (CTA) showed pericardial thickening with irregular margins and minimal effusion, without coronary stenosis. Cardiac magnetic resonance imaging (MRI) confirmed moderate pericardial effusion with significant enhancement. A diagnosis of pericarditis was established, and treatment with moxifloxacin (20-day course) and supportive therapy was initiated.

On hospital day 3, the patient developed worsening dyspnea, tachycardia (heart rate 157 bpm), and elevated inflammatory markers (WBC 19.59 × 10^9^/L, neutrophils 82.3%, procalcitonin 1.45 ng/ml). Repeat TTE revealed moderate-to-large septated pericardial effusion. Ultrasound-guided pericardiocentesis was immediately performed, yielding 540 ml of thick yellow purulent fluid over three days. Biochemical analysis demonstrated nucleated cells 128,968 × 10^6^/L (97% polymorphonuclear), lactate dehydrogenase 2,959 U/L, confirming PP. The drainage catheter was removed on day 3 due to the patient's complaint of catheter-related chest pain, diminished output, and improved dyspnea. TTE showed minimal residual effusion.

**Figure 1:**
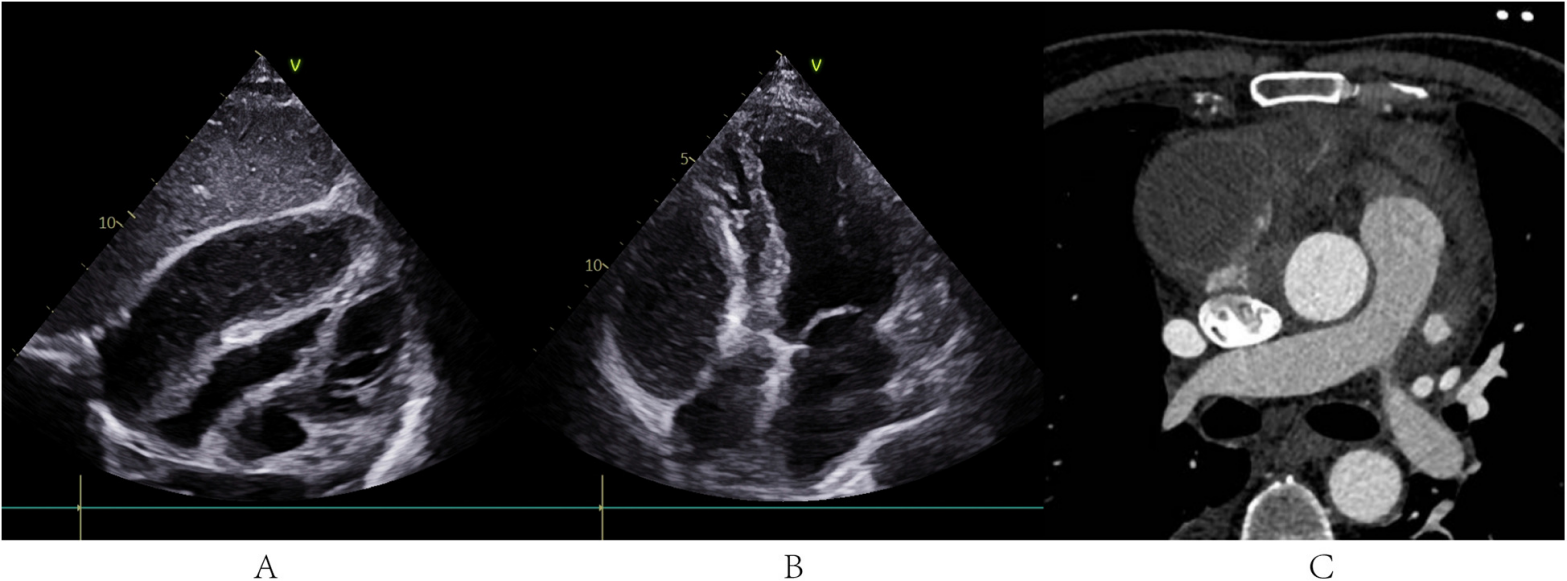
TTE and cardiac CT before PTCA guidewire-based pericardiocentesis. **(A,B)** TTE revealed a loculated gelatinous effusion in the posterior wall of the right pericardial cavity compressing the right atrium and ventricle, with pericardial adhesions. CT confirmed pericardial thickening, blurred pericardiophrenic fat planes, and a septated purulent collection adjacent to the right atrium.

During hospitalization, the patient developed lower limb venous thrombosis, atrial fibrillation, and moderate pleural effusion, which were managed with anticoagulation therapy (low-molecular-weight heparin) and targeted heart failure management. On day 17, persistent dyspnea prompted a repeated TTE, which revealed a loculated gelatinous effusion in the posterior wall of the right pericardial cavity compressing the right atrium and ventricle, with pericardial adhesions (Figures 1A,B and Supplementary Videos S1). Cardiac computed tomography (CT) confirmed pericardial thickening, blurred pericardiophrenic fat planes, and a septated purulent collection adjacent to the right atrium (Figure 1C).

**Figure 2:**
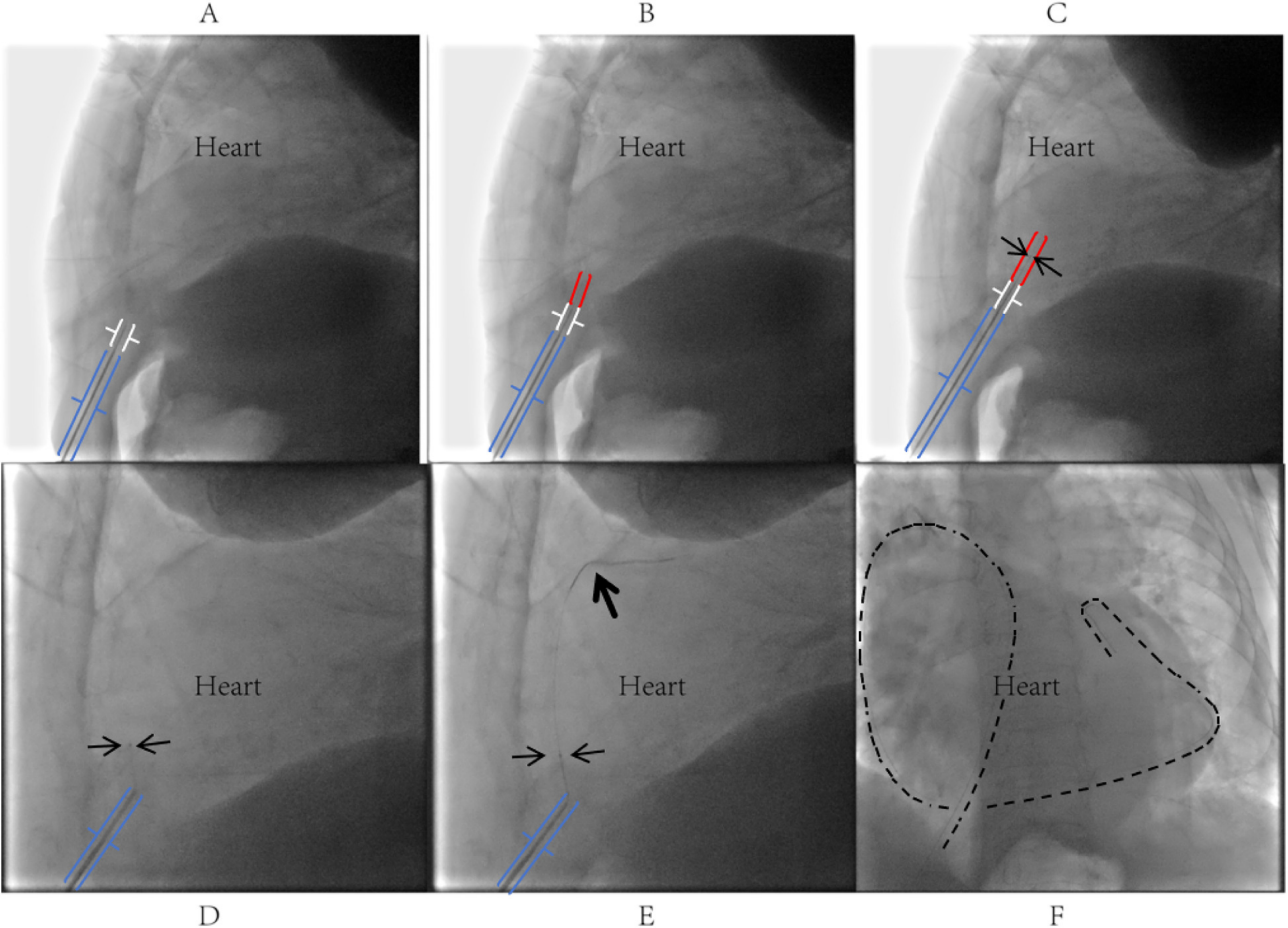
Procedural steps of PTCA guidewire-based pericardiocentesis. **(A)** Under left lateral fluoroscopic guidance, the puncture needle tip was inserted through the skin at the left costophrenic angle, traversed superficial tissues into the inferior region of Larrey's triangle (formed by the xiphoid process, right ventricle, and diaphragm), maintaining a 2–3 cm distance between the needle tip and cardiac silhouette; a blunt-tipped metal tube was slowly advanced through the needle lumen to approximate the parietal pericardium; **(B)** the stiff end of the PTCA guidewire was slowly advanced through the metal tube (MAP802 PhD Hemostasis valve, Merit Medical Systems, Inc.) to penetrate the parietal pericardium into the pericardial cavity; upon entering the pericardial cavity for approximately 1–2 cm, the guidewire typically exhibited subtle oscillations synchronized with cardiac pulsation; **(C)** a 1.8Fr Terumo Finecross microcatheter was advanced along the PTCA guidewire into the pericardial cavity and secured in place; **(D)** after withdrawing the guidewire, the soft end was reintroduced into the Terumo microcatheter and gently advanced into the pericardial cavity; **(E,F)** successful pericardial puncture was confirmed by observing the guidewire coiling along the cardiac contour, followed by insertion of a sheath or drainage catheter over the guidewire and microcatheter as clinically required.

**Figure 3:**
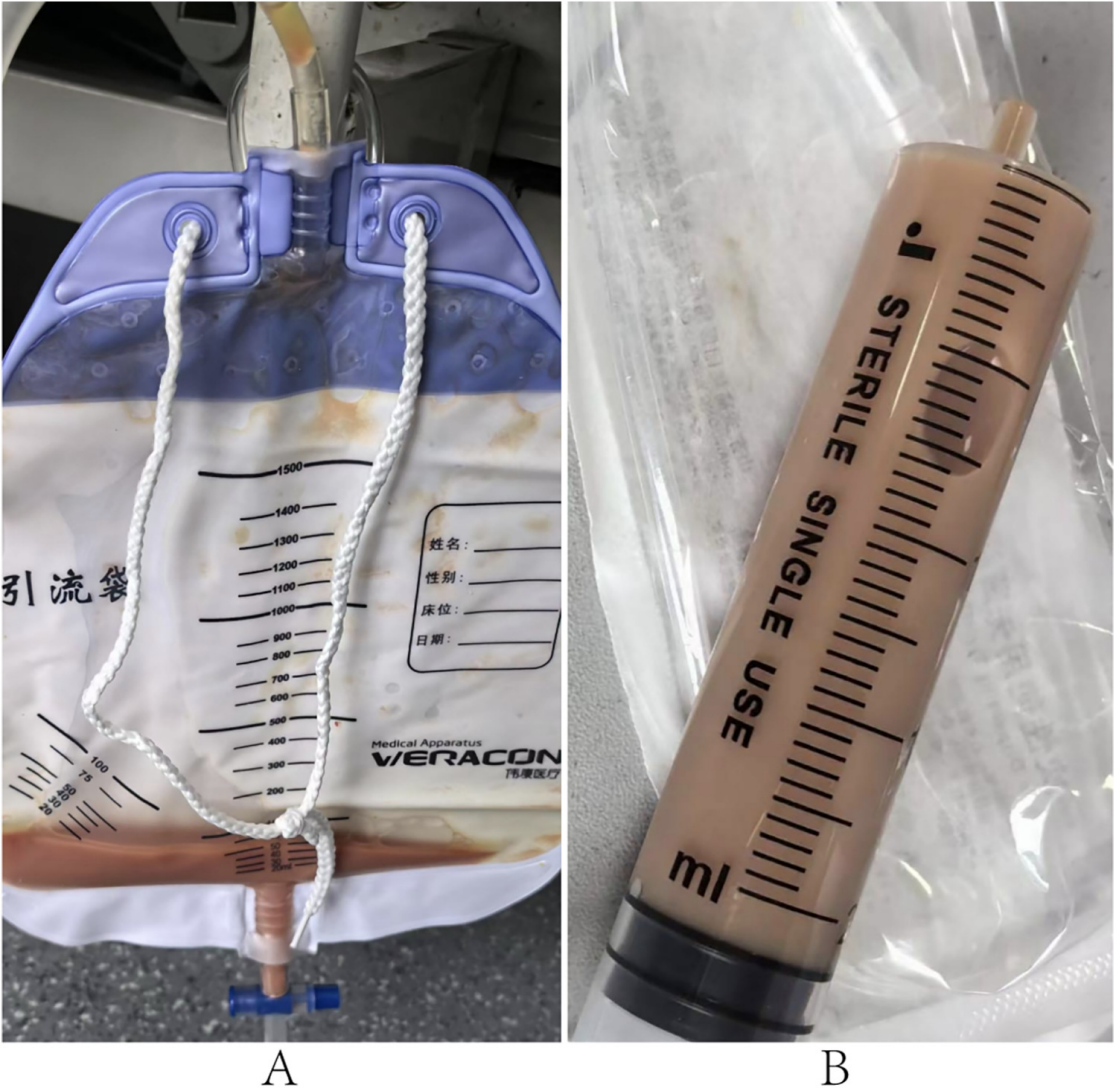
Drainage fluid 24 h after intrapericardial alteplase. **(A,B)** Show characteristics of the pericardial drainage fluid.

Given the loculated nature of the effusion with pericardial adhesions, conventional pericardiocentesis was technically challenging, while the prolonged use of low-molecular-weight heparin for lower-limb thrombosis and atrial fibrillation further increased procedural risks. Therefore, digital subtraction angiography (DSA)-guided PTCA guidewire-based pericardiocentesis was performed using a Sion guidewire and Terumo microcatheter (Figure 2). Despite aspirating 350 ml of viscous purulent fluid, the drainage catheter became nonfunctional within three days, with worsening symptoms and unchanged effusion on TTE. Reintervention involved repositioning the catheter under DSA and advancing a J-tip guidewire through the existing tract. A 6F sheath was subsequently inserted, allowing aspiration of 400 ml of purulent fluid via the side port. The sheath was then exchanged for a central venous catheter, through which 5 mg of alteplase (diluted in 20 ml of saline) was instilled into the pericardium. The catheter was clamped for 24 h to facilitate fibrinolysis, yielding an additional 400 ml of drainage over 72 h (Figure 3).

**Figure 4:**
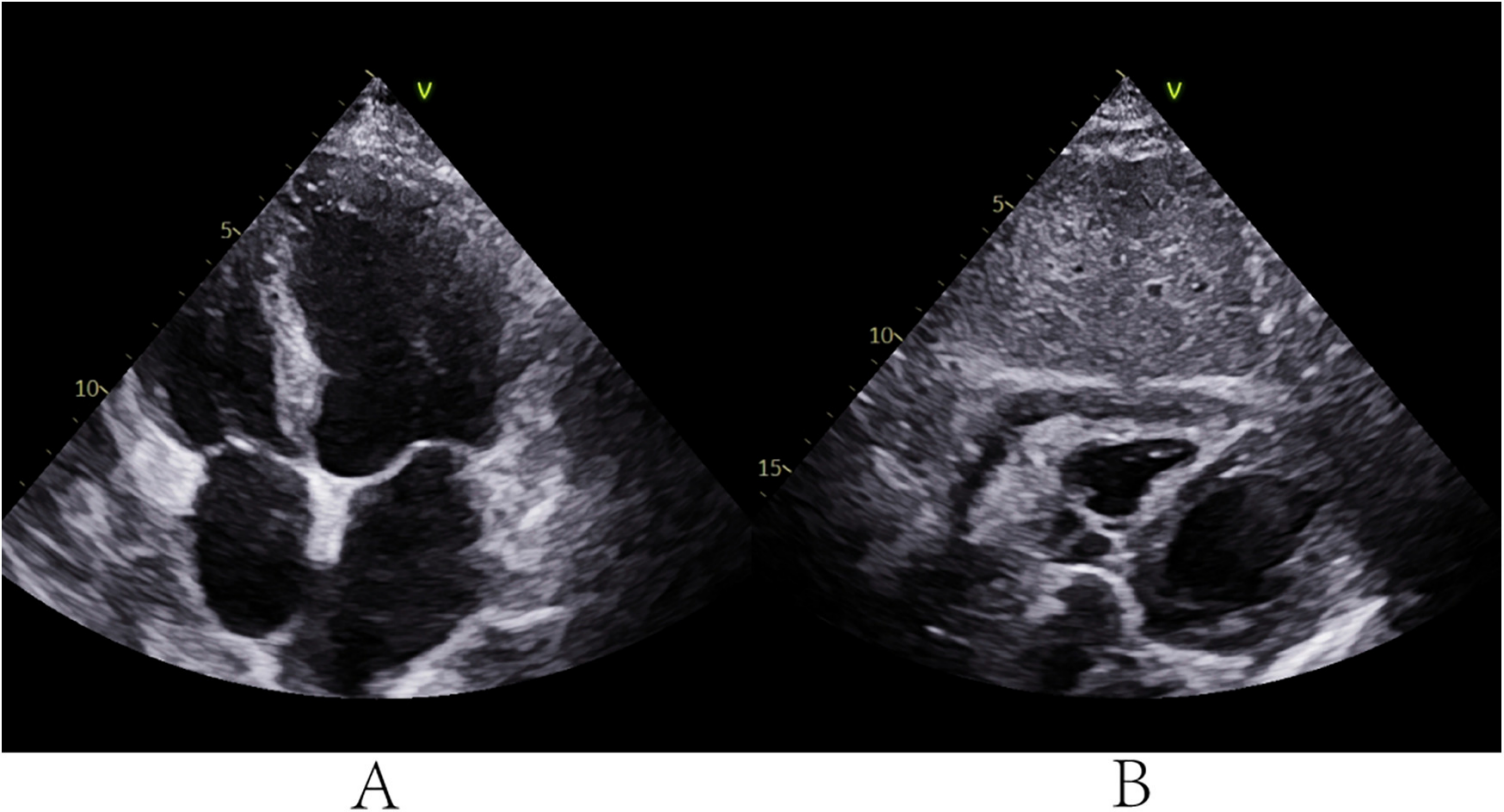
TTE on the 5th day after surgery. **(A,B)** TTE showed a small amount of pericardial effusion.

The foul odor of the aspirated pus raised clinical suspicion of anaerobic infection. Accordingly, antibiotic therapy was adjusted from moxifloxacin to cefoperazone-sulbactam combined with ornidazole, guided by postoperative cultures confirming anaerobic and Gram-negative bacilli (G-b) in the pericardial lavage fluid. The patient's symptoms gradually resolved, with TTE on postoperative day 5 showing minimal residual effusion. The catheter was removed, and intravenous antibiotics were continued (Figure 4). At the 10-month follow-up, the patient remained asymptomatic with no recurrence of pericarditis or associated symptoms (chest pain or dyspnea), and TTE demonstrated complete resolution of effusion and pericardial thickening.

We report an table of the critical events in the patient's medical history (Supplementary Table 1).

## 3 Discussion

PP may be primary or secondary to other infectious processes. In developed countries, the most common pathogens include Staphylococcus, Streptococcus, and Streptococcus pneumoniae, with associated abscesses (50%) or pneumonia (33%) being predominant predisposing conditions (2). In the present case, no extracardiac infectious foci or immunocompromised risk factors were identified, supporting a diagnosis of rare primary PP. While intravenous antibiotics and effective pericardial drainage form the cornerstone of therapy, these measures often prove insufficient due to fibrin septations creating multiloculated effusions that impede drainage and antibiotic penetration (3). Surgical interventions, such as subxiphoid pericardiostomy with pericardial cavity rinsing or manual lysis, may achieve more complete evacuation. Alternatively, intrapericardial fibrinolysis offers a less invasive approach (2).

However, experience with fibrinolytic agents in PP remains limited, with no consensus on optimal drug selection, dosing, or administration protocols. Early attempts using urokinase or streptokinase demonstrated efficacy but carried substantial risks of intrapericardial hemorrhage (4–6). Alteplase (recombinant tissue plasminogen activator, r-tPA) appears safer. A case series of four PP patients reported successful outcomes with 20 mg of alteplase in 100 ml saline administered via large-bore Pezzer drains, clamped for 24 h and repeated once if needed, without major complications (7). Other regimens, including daily 10 mg doses for three days or a single 10 mg dose in 20 ml saline, also achieved favorable results (8, 9).

In our case, the patient developed pathological features of constrictive pericarditis—including eccentric loculated effusion, visceral-parietal adhesions, and pericardial thickening—attributable to increased fluid viscosity and multiloculated septations that hindered effective drainage during the PP disease course. While surgical intervention could potentially provide effective management for such complicated effusions, the procedural risks associated with the patient's anticoagulated state and comorbidity profile outweighed the anticipated benefits. Although intrapericardial fibrinolysis offers a minimally invasive alternative to extensive surgery, TTE in this case revealed a right posterior loculated effusion with high-risk visceral-parietal adhesions at conventional puncture sites, rendering standard pericardiocentesis prone to myocardial injury.

To address this, we employed a novel approach using a blunt-tipped, highly flexible PTCA guidewire (Sion). Under DSA guidance, the puncture needle was advanced to a position 2–3 cm from the pericardium, after which the blunt-tipped metal tube (MAP802 PhD Hemostasis valve, Merit Medical Systems, Inc.) was inserted until its tip contacted the fibrous pericardium. The guidewire's distal end was then used to penetrate the fibrous pericardial layers. with the support of the blunt- tipped metal tube. Then the soft end of the guidewire exchanged into the pericardium space with a 1.8Fr microcatheter. The absence of rigid needle support and reduced forward resistance allowed controlled entry into the pericardial space, significantly lowering the risks of myocardial laceration and iatrogenic tamponade compared to traditional techniques. Post-procedural intrapericardial instillation of 5 mg of alteplase (diluted in 20 ml saline) with 24-h catheter clamping achieved effective drainage (400 ml over 72 h) without complications such as hemorrhage or allergic reactions. Combined with targeted antibiotics, this approach led to complete recovery without sequelae.

In summary, we described a rare case of primary PP with eccentric effusion managed with PTCA guidewire-based pericardiocentesis and low-dose intrapericardial alteplase. TTE demonstrated a large, eccentric, highly viscous loculated effusion, rendering conventional drainage both technically challenging and high-risk. Using DSA-guided PTCA guidewire-based pericardiocentesis combined with 5 mg of alteplase instillation, we achieved complete effusion drainage, resolution of dyspnea, and full clinical recovery.

## 4 Conclusions

Our case demonstrates that low-dose alteplase safely promotes pericardial drainage by targeting fibrinolytic activity, though larger studies are required to validate its efficacy. Furthermore, the novel PTCA guidewire-based pericardiocentesis technique demonstrates high safety and efficacy in accessing eccentric loculated pericardial effusions, making it suitable for broader application in complex clinical scenarios involving pericardial pathologies.

## Data Availability

The original contributions presented in the study are included in the article/Supplementary Material, further inquiries can be directed to the corresponding authors.
