# Supplementary material for: Case Report: Eccentric purulent pericarditis treated by PTCA guidewire-based pericardiocentesis and intrapericardial alteplase
Source: Front Cardiovasc Med. 2025 Aug 5;12:1660781. doi: 10.3389/fcvm.2025.1660781 (PMC12361241; doi:10.3389/fcvm.2025.1660781)
Supplement: Supplementary file 2 [file Datasheet1.docx]

Supplementary Material

# Supplementary Figures


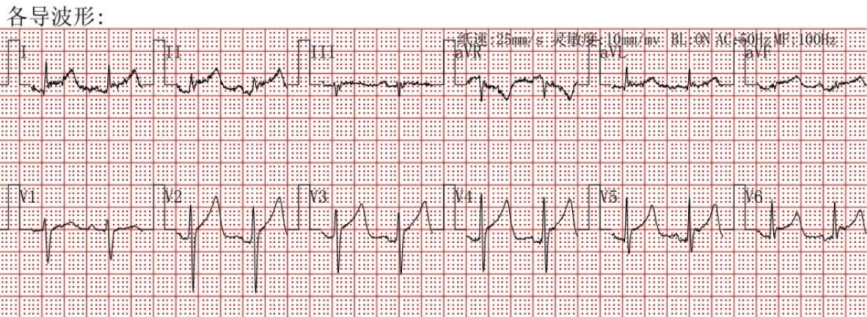


**Supplementary Figure 1.** Electrocardiography (ECG) demonstrated ST-segment elevation (0.1–0.3 mV) in the lateral, inferior, and extensive anterior walls

# Supplementary Tables

**Supplementary Table 1**

Timeline of critical events

| Timepoint | Key Event |
| --- | --- |
| Day 1 | Admission for chest pain and fever. ECG showed ST-segment elevation in multiple leads. Laboratory tests revealed leukocytosis. Coronary CTA and cardiac MRI demonstrated pericardial thickening with effusion, supporting the diagnosis of pericarditis. Initial therapy: moxifloxacin + supportive therapy. |
| Day 3 | Worsening dyspnea, tachycardia, elevated inflammatory markers. TTE: moderate-large septated effusion. Ultrasound-guided pericardiocentesis drained 540 mL purulent fluid, biochemistry confirmed PP. |
| Day 5-7 | Diagnosis of lower limb venous thrombosis, atrial fibrillation, and moderate pleural effusion. Anticoagulation therapy and targeted heart failure management. Catheter removed due to pain/diminished output. |
| Day 17-18 | Persistent dyspnea. TTE and cardiac CT revealed loculated gelatinous effusion compressing right chambers with pericardial adhesions. First DSA-guided PTCA Guidewire-Based Pericardiocentesis. |
| Day 21 | Catheter dysfunction with symptom recurrence; DSA-guided reintervention aspirated 350 mL. Intrapericardial alteplase (5 mg) instilled: catheter clamped for 24h, yielding additional 400 mL drainage. Antibiotic adjustment: cefoperazone-sulbactam + ornidazole. |
| Day 26 | TTE showed minimal residual effusion; catheter removed. |
| 10 months  later | The patient remained asymptomatic with no recurrence of pericarditis or associated symptoms (chest pain or dyspnea), and TTE demonstrated complete resolution of effusion and pericardial thickening. |
